# Factors Conditioning Sexual Behavior in Older Adults: A Systematic Review of Qualitative Studies

**DOI:** 10.3390/jcm9061716

**Published:** 2020-06-03

**Authors:** Adrián Jesús Ricoy-Cano, Esteban Obrero-Gaitán, Francisco Caravaca-Sánchez, Yolanda María De La Fuente-Robles

**Affiliations:** 1 Social Work Department, University of Jaén, 23071 Jaén, Spain; adrian.ricoy@gmail.com (A.J.R.-C.); caravaca@ujaen.es (F.C.-S.); ymfuente@ujaen.es (Y.M.D.L.F.-R.); 2 Physiotherapy Area, Department. of Health Sciences, University of Jaén, 23071 Jaén, Spain

**Keywords:** sexual behavior, sexuality, aging, physiological sexual factors, psychosocial sexual conditioning factors, qualitative research

## Abstract

The sexual behavior of older adults, especially women, has undergone changes in recent years, though there are still certain stereotypes today related to pathophysiology, beliefs, culture and tradition that negatively affect older adults’ sexual activity. The aim of our review is to present the main qualitative studies analyzing how physiological and psychosocial factors affect sexual behavior in older adults. A systematic review of these qualitative studies was carried out. All stages of this review were carried out peer-to-peer in order to guarantee minimized bias. A bibliographical search was completed between February and April 2019, in Web of Science, Scopus, PubMed Medline, PsycINFO ProQuest and CINAHL. To analyze the findings of the selected qualitative studies, a “Thematic Synthesis Analysis” was performed, using Eppi-Reviewer 4 software (UCL Institute of Education, University of London, UK). The quality of the studies was assessed with a CASP-Qualitative-Checklist. A total of 16,608 references were screened and 18 qualitative studies were included in this review. The studies involved 2603 participants across seven countries, most being women (approximately 80%). We identified a wide variety of physiological and psychological factors that can influence the sexual behavior of older adults, such as the presence of pathologies (erectile dysfunction and menopause), the strength of spiritual beliefs, and patriarchal roles upheld by upbringings conveying that women’s role is to provide men with sexual pleasure. Biological age in relation to stereotypical models of sexual behavior, emphasized as a risk factor in the contraction of sexual diseases, seems to play a relevant role as a factor limiting sexual behavior in older adults.

## 1. Introduction

According to the World Health Organization (WHO), sexual health is defined as “a state of complete physical, mental, emotional and social wellbeing related with sexuality” [1]. Sexual health is necessary for the experiencing of a full life for all people [2], especially in older adults, who represent a population group comprised of those aged 50 or over [3]. Sexuality is a multifaceted construct that encompasses sexual activity, function and behavior [4]. Sexual activity is an important marker of quality of life in older adults, associated with mental and physical well-being, satisfactory relationships, and reduced risk of chronic diseases [5]. Some recent studies have analyzed overall sexual attitudes (such as sexual desire [6] and sexual satisfaction [7]) during aging in different age groups. In this regard, it has been proposed that older adults harboring positive sexual attitudes during aging report positive sexual experiences, which may be related to other factors. In recent years, a large number of factors have been identified as possible barriers limiting the sexual function, and impeding satisfying sex lives, in older adults [8,9]. Depression, anxiety, lack of sexual reciprocity in the couple, a monotonous and repetitive sexual relationship and illness, among others, may be responsible for changes in sexual behavior in older adults [10]. In addition, the literature suggests that personal, social and cultural stereotypes can have a negative impact on the sexual behavior of older adults [11]. In this regard, to guarantee full and healthy sex lives among this population, it is essential to address a number of different factors affecting their sexual behavior.

Until recently, most research on sexuality has excluded older people as a population [12]. Older adults were often considered asexual, and the main research topics relating to sexuality focused on young people [13]. However, several studies suggest that sexual activity continues to play a fundamental role in the lives of middle-aged and older adults [14,15]. It is important to note that, although the frequency of sexual activity decreases with age, older adults are not asexual [16]. Asexual behavior is characterized by the absence of sexual attraction [17]. Several studies, however, report that older adults do experience sexual impulses and desires [18], and attach some importance to sex [19]. Although this is still true, other elements that determine sexual behavior can be identified, such as physiological and psychosocial factors [20]. Along this line, a growing body of literature on this topic has emerged, including several reviews on aging and sexuality [21,22]. In particular, recent reviews conducted by Træen et al. (2017) [23,24] explored several topics, such as sexual function, sexual difficulties, sexual satisfaction and body image in older adults. Other recent literature reviews focus on more specific topics within this area of research, like HIV/AIDS prevention [25], sexuality in institutionalized care [26] and sexual health care in old age [27]. For instance, Sinković and Towler [28] recently conducted a systematic review of the qualitative literature, including qualitative and mixed methods. They found that negative stereotypes regarding the sex lives of older adults persist, and identified two overarching thematic categories associated with these stereotypes: (1) psychological and relational aspects of sexual behavior (such as sociocultural aspects); and (2) health and sexuality (such as the effects of illness on sexuality).

There are countless physiological factors that can create barriers to sexual expression in older adults [29]. In the case of men, atrophy of the sexual organs, a decrease in testosterone levels, erectile delay and poor function, inability to maintain arousal and reduced sexual desire stand out, due to low hormone levels [30]. Factors influencing women include a decrease in estrogenic secretion after menopause, decreases in lubrication, the contraction of the cervix and uterus, the elasticity of breast tissue, breast size, atrophy of the vaginal canal, and a decrease in the size of the vagina [31]. Along this line, previous quantitative studies consistently indicate that sexual behavior in older adults is associated with important physiological factors, such as health and illness-related outcomes [32,33], men’s erectile dysfunction [34,35], women’s lubrication difficulty [34,36], the influence of menopause [37], old age [38] and distorted personal body image [24,35]. Similarly, numerous psychosocial factors that affect the sexuality of older adults are readily apparent [15]. A recent study found that the interest of older adults in sex is related to certain psychosocial factors, such as a positive perspective, openness to experimentation, perceived positive or negative relationships, the number of family members in the social network, well-being, personality traits, the characteristics of the relationship, depression, anxiety, and marital status and the length of the relationship [34]. Furthermore, quantitative studies have greatly expanded our knowledge of psychosocial factors associated with sexual behavior, such as asexual and ageist stereotypes of older adults’ sexuality [11,39], social values [39], positivity [40], and the role of religion and spiritual beliefs [24,41].

After an initial review of the literature, a higher number of physiological and psychosocial conditioning factors, affecting sexuality, modifying sexual behavior and decreasing sexual activity, were found. Other topics related to sexual behavior that have also attracted the attention of primary studies are sexual activity, satisfaction and the quality of sexual relationships [12,42], the relationship between sexual and mental health [27,43], and the beliefs and attitudes of caregivers and professionals towards sexuality in older adults [44,45]. Of all these studies, very few provide a summary of qualitative research and assess the robustness of qualitative studies [28]. The aim of this current review of qualitative studies is to gather and synthesize the evidence available on how physiological and psychosocial factors, supported by scientific evidence in recent years, can modify sexual behavior in older adults. Thus, our review question is: How can conditioning sexual factors—physiological, psychosocial, and psychological—alter or modify the sexual behavior of older adults? In addition, we delve into certain meanings and perceptions that older people assign to sexually limiting factors.

## 2. Material and Methods 

### 2.1. Protocol Review Design

A systematic peer review of qualitative studies was conducted. All the steps to carry out this review were performed by two authors, and the discrepancies were resolved by consensus or by consultation with a third researcher. This review was reported according to the Preferred Reporting Items for Systematic Reviews and Meta-Analysis (PRISMA, supplementary Table S1) guidelines [46]. In addition, Chapter 20 of the *Cochrane Handbook for Systematic Reviews of Interventions*, [47] and Chapter 2 of the *Joanna Briggs Institute Reviewers’ Manual*, [48] which provide information on how to carry out qualitative research, methodologically, were used to implement the review methods.

### 2.2. Search Strategy

The search strategy aims to find qualitative studies that examine the phenomena in question. A bibliographic search was conducted on the Health and Social Sciences databases: Web of Science, Scopus, Pubmed Medline, PsycINFO ProQuest and CINAHL (Cumulative Index to Nursing and Allied Health Literature, EBSCOhost). A search was also conducted using the reference lists of other scientific studies of interest, as well as other reviews already published to date. The final and updated bibliographic search process was carried out between February and April 2019. No language, publication date and free full-text access filters were applied. An expert in bibliographic searches on current topics was consulted to develop a proper bibliographic search strategy. After consulting the Medical Subjects Heading (MeSH) on Medline, the ProQuest and EBSCO Thesaurus, we identified as keywords the terms *“sexual behavior”*, *“physiological sexual dysfunctions”, “psychosocial sexual risk factors”, “older adults”* and *“qualitative research”.* Table 1 shows the search strategies used in the different databases, with their specific tags, and the use of the Boolean operators. An adaptation of the qualitative review of the PICO tool (Population, Intervention, Comparison and Outcome) was used to design our question review [49,50]. Specifically, we propose the use of PCO (Population, Context and Outcome) adaptation to more appropriately suit a qualitative methodology [51]. The modified PCO framework for designing our question review appears in Table 2. 

### 2.3. Study Selection

Two blinded independent reviewers (A.J.R.-C. and E.O.-G.) screened the titles and the abstracts of the full texts of the studies proposed for inclusion in this systematic review of qualitative studies. An article was examined in detail, and subsequently, if at least one of the researchers selected it during the inclusion phase, by title and abstract, a third researcher (Y.M.D.L.F.-R.) resolved possible discrepancies that arose during the full-text review. The inclusion criteria used were: (1) studies based on primary qualitative studies containing experiences, views and opinions; (2) those assessing the impact of physiological and/or psychosocial conditioning factors affecting sexual behavior; (3) those on older adults’ potential to have sex. As exclusion criteria, the authors proposed: (1) qualitative studies that did not report data on our topic, and articles that were impossible to obtain; (2) quantitative studies; (3) qualitative studies where the full text was not available; and (4) qualitative studies with samples younger than middle-aged adults.

### 2.4. Data Extraction

Two reviewers (A.J.R.-C. and E.O.-G.) independently gathered the characteristics of the participants, and the main outcome of each qualitative study selected. Data was extracted and managed on electronic files piloted and adapted before the final assessment of all the studies selected. To complete this stage of the study, the authors used a standardized data collection form, following the methodological recommendations proposed by Butler [52]. The specific information extracted from each paper was: title, authors, publication data, country, language, qualitative design used, the characteristics of the study’s population, the sample, and the main outcomes related to the physiological and psychosocial factors present in each sample. Following data extraction, discrepancies arose that were resolved by consensus, through a process of joint review between the authors.

### 2.5. Content Analysis

We used a narrative synthesis to identify the main findings of the qualitative studies included. Once the studies had been selected, a thematic analysis was carried out using the “Thematic Synthesis” protocol to identify the main themes and sub-themes related to the impact of physiological and psychosocial conditioning sexual factors, and how they affect the sexual behavior of older adults. According the recommendations of Thomas and Harden [53], “the synthesis takes shape in three stages that overlap to some degree: the free coding line by line of the findings of the primary studies; the organization of these ‘free codes’ in related areas in order to construct ‘descriptive’ themes; and the development of ‘analytical’ issues”. For each of these stages, there is a consensus in the work of academic peers on the generation and subdivision of thematic lines through the use of EPPI-Reviewer 4 software (UCL Institute of Education, University of London, UK) [54], aimed at research based on qualitative or mixed methodologies, which generates a template where all the information that the reviewers consider appropriate is stored, ordered and grouped (in codes and sub-codes/themes and sub-themes) according to the research questions and the phases of the thematic synthesis [55]. This process was carried out simultaneously and independently by the two authors (Y.M.D.L.F.-R. and A.J.R.-C.), and the discrepancies that arose were resolved by consensus.

### 2.6. Assessment of Methodological Quality of Qualitative Studies

One of the authors (A.J.R.-C.) performed the assessment of the methodological quality of the qualitative studies included, and the assessment procedure was verified by a second reviewer (F.C.-S.). The discrepancies that arose in the scoring and rating of the studies were resolved by consensus among them. Qualitative studies meeting the eligibility criteria were assessed for methodological quality. The quality appraisal of the qualitative studies included was conducted using the “CASP Qualitative Checklist” [56], which is recommended by the Cochrane Collaboration qualitative methods groups [55]. The CASP tool assessment consists of 10 questions addressing the rigor of the research methodology, and the credibility and relevance of the main findings [52]. No rating scale for this system was specifically developed. However, each item evaluated could be awarded the following qualitative scores: “Yes” (1 point), “Can’t tell” (0.5 points) or “No” (0 points) [52]. Accordingly, when we got a “Yes” in two-thirds of the sections of the CASP, this was rated as “High”; “Moderate” quality was considered when the score was between four and six “yeses”; and finally, if more than two-thirds of replies were “No”, the paper was recorded as “Low” quality, as used in previous qualitative review studies [57].

## 3. Results

### 3.1. Selection Process

**Figure 1:**
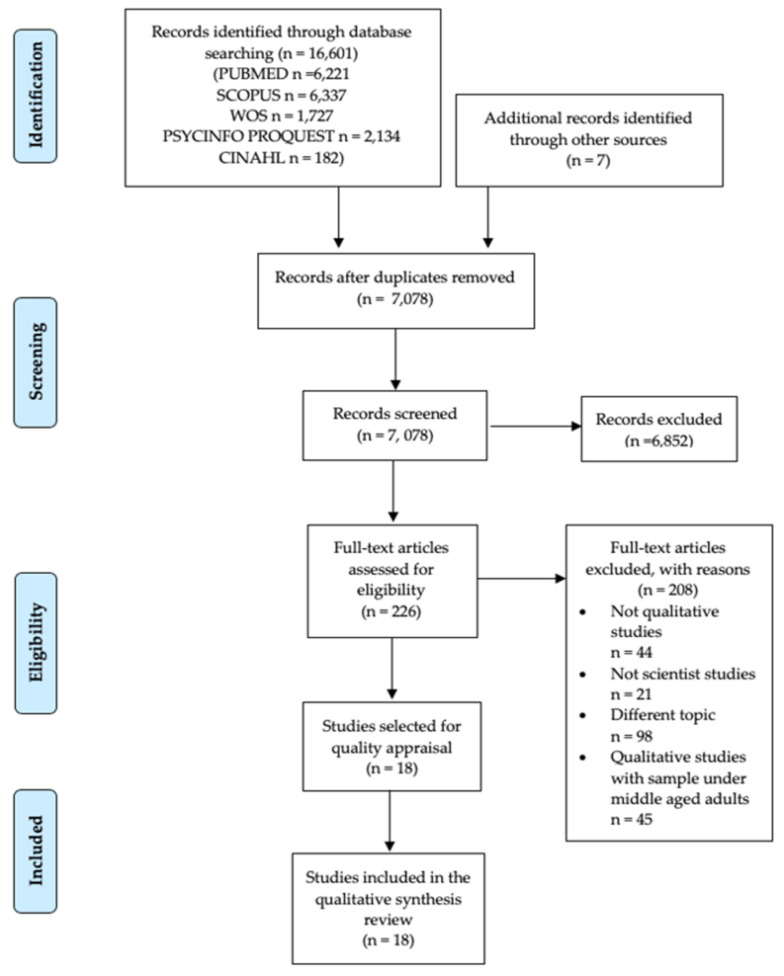
*Preferred Reporting Items for Systematic Reviews and Meta-Analysis* (PRISMA) flow chart.

The bibliographic search and study selection process is showed on the PRISMA flow chart in Figure 1. The bibliographic search yielded a total of 16,608 references. After duplicated records were removed (9530 studies), a total of 7078 references were screened. During the second stage of the screening phase, 6852 studies were eliminated after independent peer review of the title and summary, with any discrepancies being resolved by review by a third party. Additionally, in this step 23 studies were excluded due to inaccessibility of the full text of the document. A total of 226 studies was selected for full-text peer review, of which 208 references were removed after applying the proposed exclusion criteria [(1) not qualitative studies (*n* = 44); (2) not scientific studies (*n* = 21); (3) different topic (*n* = 98); and (4) qualitative studies with samples younger than middle-aged adults (*n* = 45)]. Finally, 18 studies [58,59,60,61,62,63,64,65,66,67,68,69,70,71,72,73,74,75] were included in the narrative and thematic synthesis of this systematic review of qualitative studies.

### 3.2. Characteristics of the Studies Included in the Qualitative Synthesis

Table 3 lists the 18 studies included in the review. The dates of the qualitative studies included in the synthesis span a 14-year period (2005–2019). A total of 2603 participants made up the samples, distributed across seven countries: Australia (*n* = 262), Israel (*n* = 64), the United States (*n* = 415), Turkey (*n* = 15), Spain (*n* = 729), the United Kingdom (*n* = 1103) and Iran (*n* = 15). The ages of the samples range from 50 to 90, with a mean age of 66.3 ± 4.03 years. Women constituted 80% of the sample (*n* = 2082), with men accounting for 20% in the studies included (*n* = 521). Table 3 shows the main characteristics of the qualitative studies included in our review.

### 3.3. Methodological Quality Assessment of Studies Included

In general, the methodological quality of the articles included in the thematic synthesis was Moderate (mean quality = 5.66). Seven articles exhibited High quality [58,61,64,68,69,71,74], which represents 38.89% of the total of studies; eight studies presented Moderate quality [59,60,62,63,65,70,72,75], which represents 44.44% of the total; and only three studies showed Low quality [66,67,73], which represents 16.67% of the total. Discrepant scores were shown in Items 3 and 6. Item 3 refers to the adequacy of the research design with respect to its objectives, and 15 studies [58,59,61,62,64,65,66,67,68,70,71,72,73,74,75] were inadequate as regards this criterion. Only one study [64] adequately met the requirements of Item 6, which deals with considerations in the relationship between researchers and participants. A quality assessment of the studies included is presented in Table 4.

### 3.4. Findings from Thematic Analysis 

In the following section, we provide an overview of the main findings of our review. The studies included in our review [58,59,60,61,62,63,64,65,66,67,68,69,70,71,72,73,74,75] identified three main thematic areas related to the impact of different sexual conditioning factors in older adults. Table 5 shows the main findings of our review.

#### 3.4.1. Is Sexuality Affected by Health and by Age?

Older people continue to have sexual desires and sexual interests, and enjoy an active sexual life [76]. In fact, there can be, in some cases, an increase in sexual desire and skills over the years, as subjects are progressively freed from family and work pressures [68]. Some participants associated a decrease in the frequency of sexual activity with the advent of physiological conditions, such as erectile dysfunction or menopause, among others, resulting in a loss of libido [73,74]. In general, the qualitative investigations analyzed tend to indicate that menopause significantly affects sexuality [73], and may cause a decrease in sexual desire, as well as vaginal dryness [68,72]. Other participants explained how their sexual practices and definitions of sex changed after suffering from a significant disease, such as prostate cancer. Specifically, one of the participants (male, age 77), who underwent a radical prostatectomy, shared his feelings after the operation. He expressed frustration at having to *“fiddle around to be able to get an erection”*, and that he was *“a bit sick of doing all that”* [70]. For this reason, and as stated by Syme et al., it is important to address unrealistic and socially constructed expectations about aging and sexuality, as sexual well-being is attainable but levels of functioning may vary widely [64]. For instance, a 62 year-old participant from Australia [71] stated that: *“I think there’s a bit of a stigma to older people having sex, I think people look at you oh you dirty old buggers… I think we should be saying in your 60s, 70s, 80s as long as you’re comfortable with it and you want to do it there’s nothing wrong with it”.*

#### 3.4.2. Societal Influence and Stereotypes and the Sexuality of Older Adults

Low-income, uneducated women are disproportionately taught to believe that they have an “obligation” to care for and meet the sexual needs of their husbands, and their sexual notions are often strongly influenced by religious teachings that stigmatize sex. A reduction in sexual desire has been observed in women owing to these gender stereotypes, [58,63,67] which affect their sexuality and entail a dual danger, as they induce them to discriminate based on age and gender, thereby undermining the development of their intimate relationships [66]. Indicative is the story from a sample of a Turkish woman in her 60s [74]: *“We haven’t had sexual intercourse for the last 20 years. I was not very willing previously, either. I don’t desire my husband. We are of Eastern origin. We were raised hearing “mustn’t look at men, mustn’t laugh”. That’s why I’m very strict. Sexuality never felt warm. I guess it is because we were shown so, told so”.*

When dating later in life, many traditional gender roles are evident, some of which entail courtesies towards women, such as men being the ones to ask for dates, opening doors for women, etc. Some aspects have changed; it is no longer common for men to pick up women at home, and men and women tend to meet at the place set for their date, thus breaking with the traditional gender “script” according to which the man picks up the woman [59]. Many of these traditional roles end up affecting sexuality among those of advanced ages. For women, and not necessarily for men, affection is essential to sexuality and to satisfaction with life, which women tend to associate with gender equality [63].

#### 3.4.3. Factors Impacting Sexuality in the Elderly Associated with the Study

A series of secondary issues appear in the study which are significant for a holistic understanding of the phenomenon of sexuality in the aging. Among them, we may highlight the need to differentiate between men and women in terms of sexual satisfaction, the search for new forms of sexual pleasure outside approaches based on exclusively male satisfaction, self-awareness of one’s body and personal tastes in order to discover autoerotism, self-adaptation of the older female body in relation to but not outside the framework of marriage, and sexual repression rooted in religious and spiritual beliefs, which impacts women more and has been linked to low levels of education [63,70,73]. Regarding sexual pleasure, the study shows how traditional ideals centering on male pleasure are beginning to be rejected. Participants from studies included reflect on the low levels of education they received about sex and sexual pleasure. For some, sexual pleasure is related to union, intimacy and closeness, while for others orgasm is the main form of pleasure [70,71].

## 4. Discussion

The aim of our review was to gather several qualitative studies, in order to identify how physiological and psychosocial conditioning factors affecting sexuality can induce changes in the sexual behavior of older adults, as well as to highlight the sub-themes that appear jointly in the research on this thematic base. From the selection of studies conducted, the aim was to offer an assessment of the quality of the research and a thematic synthesis, in order to portray what and how qualitative research on sexuality among older adults is being conducted. As a result, useful tools can be suggested, and new lines of research can be opened up.

Contrary to many beliefs and stereotypes about sexuality among older adults, this study attempts to demonstrate that sex actually constitutes a significant aspect of the lives of many older adults, and that it can be enjoyed in a satisfactory and active way [73,77]. However, we must recognize that the appearance of diseases is commonly associated with sexual dysfunction in both men and women. Sicknesses in older adults often lead to anxiety, marital discord and withdrawal, [73,77,78] which can lead to alterations in sexual behavior and relationships. Along these lines, it is necessary to clarify that fragility is not, in itself, a barrier to sexuality [79].

Some of the study’s participants believe that changes in sexual health and age are conceptually inseparable [61]. Regarding physiological factors that affect sexuality, menopause among women and erectile dysfunction among men are the most frequently discussed [68,74]. Among women, our findings are in line with previous studies reporting the negative impact of menopause on women’s ability to engage in and/or enjoy sexual activities [80]. However, prior authors have found that interpersonal factors clearly play an important role in menopause, reducing levels of distress and increasing sexual desire [81]. Regarding erectile dysfunction, studies support a correlation between age, erectile dysfunction and loss of sexual desire [82], adversely affecting quality of life. [83] However, more recent authors have found that the negative effects of erectile dysfunction on sexual activity can be mitigated by professional help and social support from relatives to remain sexually active [84,85]. Similarly, an association between sexual dysfunction and emotional problems derived from erectile disfunction may be observed [19,60].

With increasing age, a series of changes occur in female genital physiology that can cause a loss of sexual desire, problems with vaginal lubrication and orgasmic disorder [86]. This is because, with age, the proportion of smooth muscles/connective tissue in the clitoris and vagina increases in favor of connective tissue. Atrophy also occurs in the smooth muscles of the vaginal wall. The capacity of the clitoris to harden, and the ability of the vagina to expand, is greatly impaired, and vaginal secretions decrease [87].

In this review, we attempt to uncover and dispel some of the stereotypes and false beliefs that are widely held regarding sexuality in older people, based on the evidence found. Mention is made of the existence of “sexual scripts” and “gender roles” that affect both the social and sexual status of older adults, particularly impacting women [63]. Asexuality among older people is a complex issue to evaluate; while various studies examine the desires and sexual interests of older people from a variety of angles [19,88,89,90,91,92], other studies emphasize that asexuality in older women can still be found [88,93]. Thus, due to conflicting findings, it seems necessary to carry out future research that explores asexuality and its association with more physiological and psychosocial factors [94].

Within the findings of this study, sexual satisfaction emerges as a key sub-theme. As in the case of asexuality, the evidence found does not allow for simple conclusions. In the study conducted in Spain by Freixas [73], the sex lives of the female interviewees were satisfactory. However, other studies indicate that satisfaction and desire are an intrinsic part of female behavior, but are not satisfied in marriage [62,69]. These results are partly affected by the limitations of this review, and a compendium of analyses covering the themes of “asexuality” and “sexual satisfaction” in greater depth would yield more significant evidence.

Regarding trends in individuals’ autoerotic behavior, sexual self-stimulation, according to one of the studies analyzed, based on an examination of sexuality in older Spanish women, decreased after the age of 70. Differences are also seen in terms of the age of the onset of masturbation between lesbian and bisexual women, compared to heterosexuals: 33.33% of lesbian and bisexual women stated that they began these practices in childhood, compared to 15.6% among heterosexual women. 59.95% of the participants in this study admit to masturbating frequently or occasionally. Within these sections, there are also slightly higher rates of autoerotic activity among lesbian and bisexual women [73]. This information can be verified against that of other studies, indicating that the practice of masturbation continues even after age 70, this being one of the most common ways in which older women find pleasure. No statistically relevant differences were found regarding masturbation between the 50–59 year-old group and the 60 year-old and older group [95]. Further research in the field of autoeroticism in older women is required, in order to carry out more detailed analyses and draw conclusions about behaviors and their frequencies.

Nuances were sought within the concept of global sexual satisfaction, as is the case with pleasure. What was found in this study is that contemporary society seeks to break with traditional precepts centering on male pleasure, while simultaneously demanding higher levels of education regarding sexuality, health and sexual security, and sexuality during aging. These higher levels, however, are rarely achieved [68,70,71,72].

For this review we have not sought to ignore the influence that spirituality and religion may have on sexuality among older adults. These are factors key to comprehending the sexual attitudes of many older adults, as they shape sexual behavior and even how satisfaction is viewed in line with such religious beliefs. [63]. In fact, prior authors found that some religion-dominated cultures provide a dark, immoral image of sexual desires, while others allow sexual freedom, [18] religion being an important factor shaping sexual understanding, sexual education and public health [59].

An effort has been made to conduct a review of qualitative studies to assess which factors fundamentally shape sexuality among older adults. Using thematic synthesis, it was possible to highlight evidence, as well as conflicting views, regarding the themes analyzed. It can be fairly stated that there is a range of physiological and psychosocial factors that influence sexual behavior during old age. Some studies have found that their participants consider a better understanding of sexuality to be of value—through sex education, for instance.

This qualitative review has a number of limitations. Firstly, the topical exclusion of themes that may prove to be of great interest for future research, such as the physiological benefits of sexual relations for older adults, the beliefs and attitudes of caregivers and healthcare professionals towards sexuality in older adults, and issues related to the trauma of sexual abuse [96] and its relationship with sexual dysfunction [97]. The dearth of literature on the issue of sexual activity among older adults has hindered the thematic synthesis process. Secondly, in some cases, several studies were taken from the same author, which, despite being different research studies, retain some of the same features (location, target population, design/method of data collection and sample ages) [60,68,69,70,71,72,75], which limits one’s ability to generalize from their results. Third, this review analyzed studies with older adults from different geographical areas, including Europe [73,74,75], Asia [63], Australia [68,69,70,71,72] and the USA [61,64]. Thus, the results must be interpreted as accounting for heterogeneous socio-cultural contexts and belief systems. Future reviews are needed that exclusively feature participants from similar geographical areas, in order to avoid social heterogeneity issues. Fourth, most of the qualitative studies included in this review focus on heterosexual relationships. Brief mentions are made in some studies of the fantasies and mental representations that heterosexual people make that include homosexual elements [70], or desire in lesbian women [73]. It may be valuable, in future studies, to analyze how social pressures influence the sexual behavior of older homosexual people. Fifth, the sample was composed mostly of women (approximately 80%), and only across seven countries. As such, our findings are limited in terms of their potential extrapolation to older adults, including typical cross-cultural, international comparisons from a larger number of countries and different cultural contexts [98], such as those by Winn and Newton, [99] who investigated sexual attitudes and behaviors from over 100 different cultures, and Laumann and colleagues [100], who investigated sexual attitudes and behavior in 29 countries. Nevertheless, the findings presented here might be applied to contexts in other countries. The results of the study should be treated with caution in the case of males. The sex ratio (5:1) of this study shows clear evidence for the female sex. However, some topics are explored through qualitative analysis, and certain experiences and perceptions of the men interviewed may be useful for understanding some factors considered to be limiting sexuality. Thus, more quantitative and qualitative studies are needed—or larger samples of men, and more countries. Finally, this review focuses only on the analysis of qualitative studies. Future studies may collect results on the social meanings and realities presented in this study and approach them from quantitative or mixed approaches. The findings of this research review facilitate a synthesis of the main aspects related to sexuality during late adulthood, which may be used to shape policies regarding public health, education, the promotion of sexual health, and an active and healthy lifestyle during late adulthood [68,74]. There is a need to understand the sexual status of older adults, and forego value judgments and associated stereotypes. The results found in this review, regarding knowledge of the physiological and psychosocial factors affecting sexuality in older adults, are valuable for the execution of future research, especially in finding an association between the frequency of sexual relations, age, and the associated factors mentioned.

Similarly, certain real experiences of older people are looked at, including the way they understand sexuality, their current sexual status, their satisfaction, desires, self-erotic habits, the importance they attach to sex during late adulthood, and the influence of their religious beliefs. This review may be useful for any expert or individual interested in the subject of “sexuality in older adults”.

## 5. Conclusions

As the results of our review show, a set of socially assigned beliefs and attributes appear, associated with age, which, while they do not completely determine, certainly limit the behavioral expressions of older adults’ personal sexuality. In addition to these social influences, age plays a determining role via the contraction of sexual diseases and sexual infections. Older people, and especially women, continue to have sexual desires and interests, and may enjoy satisfying and active sexual lives. To increase knowledge about sex, education is required for better understanding sexual needs during this life stage, and for being able to enjoy sexual activity in a safe and comprehensive way.

Findings from this qualitative review show that the most common physiological and psychosocial conditioning factors that affect sexuality, and that can induce alterations in sexual behavior in older adults, are the influences of menopause, erectile dysfunction, older age, stereotypes and religion. Thus, our findings suggest that a number of topics should be considered by future quantitative researchers interested in sexual behavior among older adults. Moreover, future research may help us understand which physiological and psychosocial factors are negatively or positively associated with sexual behavior, to improve future research on aging and sexuality, to develop educational and assistance strategies to improve sexual health, and to devise sex-related social interventions and services for older adults.

## Figures and Tables

**Table 1:** Bibliographic search strategy in each database.

Database	Search Strategy
Medline	(older adult* [tiab] OR middle aged [mh] OR middle aged [tiab] OR aged [mh] OR aged [tiab] OR aged, 80 and over [mh] OR aged, 80 and over [tiab] OR elderly [tiab]) AND (sexual behavior [mh] OR sexual behavior [tiab] OR sexual behavior [tiab] OR sex behavior [tiab] OR sexuality [tiab] OR sexual activity [tiab]) AND (sexual dysfunction, physiological [mh] OR sexual dysfunction, physiological [tiab] OR physiological sexual dysfunctions [tiab] OR physiological sexual conditioning factors [tiab] OR psychosocial sexual conditioning factors [tiab] OR psychological sexual dysfunction [tiab] OR psychological sexual risk factor* [tiab] or physiological [tiab] or psychological [tiab] or psychosocial [tiab])
Scopus	[TITLE-ABS-KEY (“older adults” OR “middle aged” OR “aged” OR “elderly” ) AND TITLE-ABS-KEY (“sexual behavior” OR “sexual behaviour” OR “sex behavior” OR “sexual activity” ) AND TITLE-ABS-KEY (“physiological sexual dysfunction” OR “physiological sexual conditioning factors” OR “psychological sexual conditioning factors” OR “psychological sexual risk factors” OR “physiological” OR “psychological” )]
Web of Science	TOPIC: [(*older adults* OR *elderly* OR * middle aged* OR * aged* OR *aged, 80 and over* OR *elderly*) AND (*sexual behavior* OR *sexual behaviour* OR *sex behavior* OR *sexual activity*) AND (*physiological sexual dysfunctions* OR * physiological sexual conditioning factors* OR *psychosocial sexual conditioning factors* OR *psychological sexual risk factors* OR *psychological sex factors* OR *physiological* OR *psychological* OR *psychosocial*)]
PsycINFO ProQuest	[AB(older adults) OR SU(middle aged) OR AB(middle aged) OR SU(aged) OR AB(aged) OR SU(aged, 80 and over) OR AB(aged, 80 and over) OR AB (elderly)) AND (SU(sexual behavior) OR AB(sexual behavior) OR AB(sex behavior)) AND (SU(sexual dysfunction, physiological) OR AB(sexual dysfunction, physiological) OR AB(physiological sexual dysfunctions) OR AB(physiological sexual conditioning factors) OR AB(psychological sexual dysfunctions) OR AB(psychological sexual risk factors)]
CINAHL	(AB older adults OR AB middle aged OR AB aged OR AB elderly) AND (AB sexual behavior OR AB sexual behaviour OR MH sexuality OR AB sexuality) AND (MH sexual dysfunctions OR AB physiological OR AB psychological)

* Pubmed Medline specific truncation in bibliographic search strategy

**Table 2:** Population, Context and Outcome (PCO) framework of our research.

Population	Older Adults
Context	Physiological and psychosocial sexual conditioning factors
Outcome	Sexual behavior

**Table 3:** Characteristics of the qualitative studies included in the review.

Author and Year	Place	Target Population	Data Collection Design/Method	Sample Ages	Size and Gender
Ayalon et al. 2019	Israel	Older men and women	In-depth interviews	60 and over	Total = 47
					23–F
					24–M
Dickson et al. 2005	United States	Older adults and married women	Depth interviews	62–79	15–F
Fileborn et al. 2015	Australia	Australian women married/with partner	Qualified semi-structured interviews	55–81	43–F
Fileborn et al. 2015	Australia	Older women single at the time of the interview	Qualified semi-structured interviews	55–81	58–F
Fileborn et al. 2017	Australia	Older men	Qualified semi-structured interviews	60 and over	27–M
			Conducted by: Phone, Skype and Face to Face		
Fileborn et al. 2017	Australia	Older men and women	Qualified semi-structured interviews	60 and over	Total = 53
					23–F
					30–M
Fileborn et al. 2018	Australia	Older men and women	Qualified semi-structured interviews.	60 and over	Total = 53
			Conducted by: Phone (*n* = 41), Skype (*n =* 10) and Face to face (*n =* 2)		23–F
					30–M
Freixas et al. 2015	Spain	Older married women	Discussion groups	50–80	729–F
			Open-closed semi-structured interviews		
Gledhill et al. 2014	Australia	Older men and women	In-depth interviews	65–84	Total = 8
			Interviews recorded in audio		2–F
					6–M
Hinchliff et al. 2008	United Kingdom	Older married women	In depth interviews	50 and over	19–F
Hinchliff et al. 2018	United Kingdom	Older men and women	Extraction of qualitative and qualitative data by application of SRA-Q	50–90	Total = 1084
					680–F
					404–M
Jen. 2017	United States	Older married women	Semi-structured interviews	57–93	13–F
Kasif et al. 2017	Israel	Widows (women)	Semi-structured interviews	62–91	17–F
			In-depth interviews		
			Face to face interviews		
Ravanipour et al. 2013	Iran	Older married women	Individual interviews	60 and over	15–F
Syme et al. 2019	United States	Older men and women	Semi-structured interviews	50 and over	373–F
Thorpe et al. 2015	Australia	Older married women	Semi-structured interviews	55–72	20–F
			Face to face interviews		
Watson et al. 2017	United States	Older married women	In-depth interviews	64–77	14–F
			Face to face interviews		
Yıldırım et al. 2018	Turkey	Older married women	Qualitative semi-structured interviews	60 and over	15–F
			Face to face interviews		

Abbreviations: F = Female; M = Male; SRA-Q = Sexual Relations and Activity Questionnaire.

**Table 4:** CASP *(Critical Appraisal Skills Programme)* qualitative checklist scores for the methodological quality assessment of the qualitative studies included.

Study	Item 1	Item 2	Item 3	Item 4	Item 5	Item 6	Item 7	Item 8	Item 9	Item 10	Score	Classification of Quality
Ayalon et al. 2019	Y	Y	N	Y	Y	N	Y	Y	Y	Y	8	High
Dickson et al. 2005	Y	Y	N	Y	Y	N	N	N	N	N	4	Moderate
Fileborn et al. 2015	Y	Y	N	Y	Y	N	N	Y	Y	Y	7	High
Fileborn et al. 2015	Y	Y	Y	Y	Y	N	N	Y	Y	N	7	High
Fileborn et al. 2017	Y	Y	N	N	Y	N	Y	Y	N	Y	6	Moderate
Fileborn et al. 2017	Y	Y	N	Y	Y	N	Y	Y	Y	Y	8	High
Fileborn et al. 2018	Y	Y	N	Y	N	N	N	Y	Y	Y	6	Moderate
Freixas et al. 2015	Y	N	N	Y	N	N	N	N	N	N	2	Low
Gledhill et al. 2014	Y	N	N	Y	Y	N	Y	Y	Y	Y	7	High
Hinchliff et al 2008	Y	Y	N	N	Y	N	Y	N	Y	Y	6	Moderate
Hinchliff et al. 2018	Y	N	Y	Y	N	N	Y	Y	Y	N	6	Moderate
Jen. 2017	Y	Y	N	Y	Y	N	Y	N	Y	Y	7	High
Kasif et al. 2017	Y	Y	N	N	N	N	Y	Y	Y	N	5	Moderate
Ravanipour et al. 2013	Y	Y	Y	N	N	N	Y	N	N	N	4	Moderate
Syme et al. 2019	Y	Y	N	Y	N	Y	Y	Y	Y	Y	8	High
Thorpe et al. 2015	Y	Y	N	Y	N	N	Y	Y	Y	N	6	Moderate
Watson et al. 2017	N	Y	N	N	N	N	Y	N	Y	N	3	Low
Yıldırm et al. 2018	Y	N	N	N	N	N	Y	N	N	N	2	Low

Abbreviations: Y = Yes; N = No; Item 1 = Clear statement of aim; Item 2 = Appropriate qualitative methodology; Item 3 = Appropriate research design; Item 4 = Sampling; Item 5 = Data collection; Item 6 = Researcher reflexivity; Item 7 = Ethical consideration; Item 8 = Appropriate data analysis; Item 9 = Clear statement of findings; Item 10 = Research value.

**Table 5:** Main findings of the thematic synthesis.

Descriptive Themes	Analytical Focus
(A) Physiological factors affecting sexuality	Theme 1. Is sexuality affected by health and age?
Stage in life	
Influence of menopause	
Erectile dysfunction	
Old age, body image and well-being	
(B) Psychosocial factors affecting sexuality	Theme 2. Societal influence and stereotypes of the sexuality of older adults
Sociocultural influences	
Beliefs about asexuality among older adults	
Stereotypes of sexuality during old age	
Professional guidance	
Singleness and sexuality in old age	
Religion and sexuality	
(C) Sub-themes that were studied	Theme 3. Factors impacting sexuality among older adults associated with the research
Sexual satisfaction	
Sexual pleasure	
Sexual desire	
Autoerotism	
Older people talk about sexuality	

## Supplementary Materials

The following are available online at https://www.mdpi.com/2077-0383/9/6/1716/s1, Table S1: PRISMA 2009 Checklist.
